# Supplementary material for: Data sharing and collaborations with Telco data during the COVID-19 pandemic: A Vodafone case study
Source: Data Policy. 2021 Oct 22;3:e33. doi: 10.1017/dap.2021.26 (PMC8649407; doi:10.1017/dap.2021.26)
Supplement: Supplementary file 1 [file dapsup.zip › S2632324921000262sup001.docx]

Thank you for all your comments on this paper, they have been extremely helpful in making it as relevant as possible for the publication. All of the comments have been addressed in the revised version that is being uploaded with this submission, but some outstanding points are outlined below:

1. Due to constraints with data retention periods some of the data is no longer accessible, which means we could not re-test and add information about the top 3 cells assumption
2. We needed a single point for the radius of gyration so we used the top cell. For the previous analysis we wanted to ensure we captured night locations broadly to avoid counting movements during the day erroneously (e.g. people did not move but the night cell wasn't captured in the top cell so it would be counted as a movement). For the radius of gyration this is not as relevant because we will be calculating actual distances and thus looking at their evolution throughout time.
3. The dip in the KPI on figure 3 on the 8th March 2020 corresponds to a set of missing data for that period due to technical error
4. We did not find significant differences between the demographics in our customer base and the general population. The age and gender are extracted from Vodafone customer information, as they need to be allocated to each individual prior to the analysis being performed

We hope this revised version addresses all comments in a satisfactory manner, and look forward to the publication
